# Increased Histone Deacetylase Activity Involved in the Suppressed Invasion of Cancer Cells Survived from ALA-Mediated Photodynamic Treatment

**DOI:** 10.3390/ijms161023994

**Published:** 2015-10-10

**Authors:** Pei-Tzu Li, Yi-Jane Tsai, Ming-Jen Lee, Chin-Tin Chen

**Affiliations:** 1 Department of Biochemical Science and Technology, National Taiwan University, Taipei 106, Taiwan; E-Mails: peitzuli@gmail.com (P.-T.L.); jane19841114@hotmail.com (Y.-J.T.); 2 Department of Neurology, National Taiwan University Hospital, 7, Chung-Shan South Road, Taipei 100, Taiwan

**Keywords:** histone acetylation, histone deacetylase, invasion, mitochondria, metalloproteinase 9, paternally expressed gene 1

## Abstract

Previously, we have found that cancer cells survived from 5-Aminolevulinic acid-mediated photodynamic therapy (ALA-PDT) have abnormal mitochondrial function and suppressed cellular invasiveness. Here we report that both the mRNA expression level and enzymatic activity of histone deacetylase (HDAC) were elevated in the PDT-derived variants with dysfunctional mitochondria. The activated HDAC deacetylated histone H3 and further resulted in the reduced migration and invasion, which correlated with the reduced expression of the invasion-related genes, matrix metalloproteinase 9 (MMP9), paternally expressed gene 1 (PEG1), and miR-355, the intronic miRNA. Using chromatin immunoprecipitation, we further demonstrate the reduced amount of acetylated histone H3 on the promoter regions of MMP9 and PEG1, supporting the down-regulation of these two genes in PDT-derived variants. These results indicate that HDAC activation induced by mitochondrial dysfunction could modulate the cellular invasiveness and its related gene expression. This argument was further verified in the 51-10 cybrid cells with the 4977 bp mtDNA deletion and A375 ρ^0^ cells with depleted mitochondria. These results indicate that mitochondrial dysfunction might suppress tumor invasion through modulating histone acetylation.

## 1. Introduction

5-Aminolevulinic acid-mediated photodynamic therapy (ALA-PDT) has been used to treat precancerous lesions and cancers [1,2]. ALA itself is not a photosensitizer, but could be converted into a natural photosensitizer, protoporphyrin IX (PpIX), in mitochondria. Therefore, exogenous ALA administration leads to the accumulation of PpIX and induces cellular damage mainly to the mitochondria after light irradiation [3,4]. Singlet oxygen is the major cytotoxic agent responsible for PDT-induced cellular damage and death [1]. It has been reported that ALA-PDT is well tolerated by patients and can be applied repeatedly without cumulative toxicity or serious side effects [5,6]. However, due to the limited light penetration, certain amounts of tumor cells might recover from PDT-induced damage and survive. We previously found that cancer cells survived from ALA-PDT have reduced mitochondrial function and metastatic potential [7]. Further studies showed that these phenotypic changes and reduced migration ability could be transmitted to their progeny, which retains the reduced function of mitochondria [7], suggesting epigenetic modifications might be involved in regulating these phenomena.

In spite of the well-known role in generating ATP, mitochondria are involved in controlling cellular differentiation, growth and death [8,9]. The mitochondrian genome contains about 4977 base pair, which encodes the major components (Complex I, IV, and V) of the electron transport chain. Most of the mitochondrial proteins are encoded in the nuclear genome. Retrograde signaling has been proposed to explain how the extra-mitochondrial factors could modulate the expression of nuclear genes encoding mitochondrial proteins, which might influence numerous cellular processes in both normal and pathophysiological conditions [10]. Retrograde signaling might be mediated by the mitochondrial membrane potential, generation of reactive oxygen species (ROS), and changes in Ca^2+^ flow [11]. Mitochondrial damage and subsequent dysfunction have been implicated in several human diseases, such as metabolic disorders, cardiac dysfunction, cancers, and aging [9,12,13,14]. Compared to normal cells, mitochondria deletion is more prevalent in tumor cells [15,16,17,18,19], suggesting mitochondrial dysfunction might relate to the progression of tumorigenesis. However, it is still not clear how mitochondrial retrograde signaling controls gene expressions in the nucleus and further regulates the carcinogenesis.

DNA methylation and histone modification are two important epigenetic mechanisms involved in regulating gene expression [20,21]. Numerous studies indicate that changes in chromatin structure by histone modifications play an important role in regulating gene transcription by affecting chromatin accessibility. Histone acetylation loosens the condensed chromatin and renders the transcriptional machinery easily to access the target DNA to enhance gene expression [22,23]. In contrast, deacetylation of histones induces more chromatin condensation and prevents the binding of DNA and transcriptional factors, which leads to transcriptional silencing. The acetylation level of core histone was regulated by histone acetyl transferases (HATs) and histone deacetylases (HDACs) [24]. It has been shown that epigenetic regulation plays an important role in modulating the gene expression profile of nuclear genome in cancer cells with depleted mitochondria (ρ^0^ cells) [25,26]. However, the cellular mechanism involved is still not clear.

In this study, we show that cancer cells with reduced mitochondria function have decreased level of histone acetylation, which are associated with the reduced migration/invasion ability and expression of invasion-related genes. Both the decrease of acetyl-coenzyme A (acetyl-CoA) content and the increase of HDAC activity are responsible for the deacetylation of core histones. Our findings suggest that histone deacetylation is crucial in the retrograde signaling induced by mitochondrial dysfunction, and responsible for the down-regulated expression of the invasion-related genes, matrix metalloproteinase 9 (MMP9), paternally expressed gene 1 (PEG1), and miR-355, the intronic miRNA of PEG1.

## 2. Results and Discussion

### 2.1. PDT-Derived A375 Variants Contain Elevated HDAC (Histone Deacetylase) Activity and Hypoacetylated Histones

**Figure 1:**
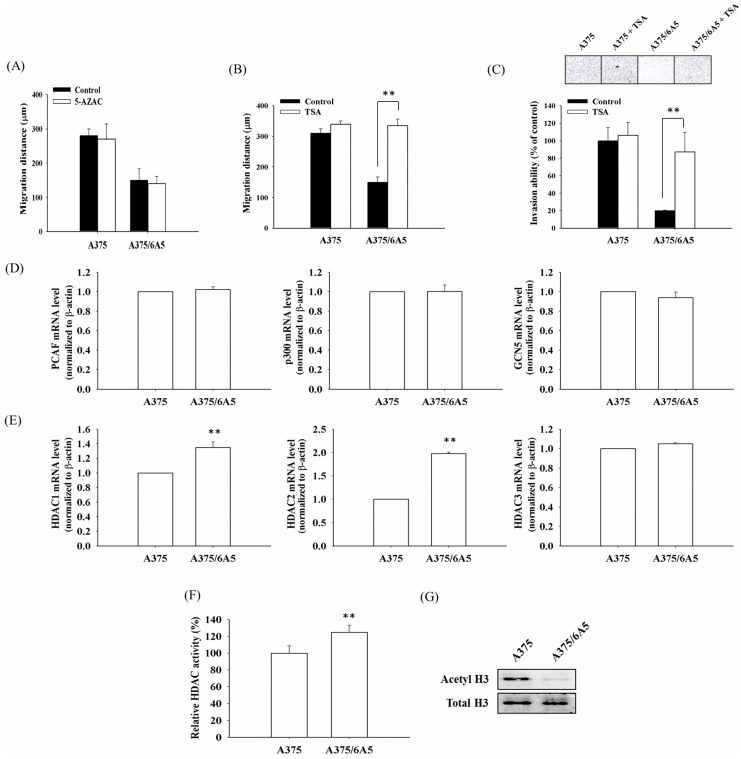
The reduced migration and invasion of PDT-derived variants resulted from the elevated expression and activity of histone deacetylase (HDAC). The scratch wound assay was used to examine the relative migration ability of PDT-derived A375/6A5 variants. The migration distance (µm) was measured 24 h incubated with or without 5 µM 5-AZAC (**A**) or 100 nM TSA (**B**); (**C**) The Matrigel invasion assay demonstrated the relative invasiveness of PDT-derived A375/6A5 cells with or without TSA treatment compared to that of parental A375 cells (200× magnification); reverse transcription PCR (RT-PCR) was used to determine the mRNA level of p300, PCAF, GCN5 (**D**), HDAC1, HDAC2, and HDAC3 (**E**) in the A375 and A375/6A5 cells. β-actin was used for normalization; (**F**) HDAC activity in the nuclear lysates of A375 and A375/6A5 cells was determined using a HDAC assay kit; and (**G**) Western blot analysis shows the levels of acetylated histone H3 in A375 and A375/6A5 cells. **, *p* < 0.01.

Previously we have shown that A375/6A5 variants, derived from human melanoma A375 cells under consecutive photodynamic treatment, have reduced mitochondrial function and cell migration and invasiveness [7]. Furthermore, the impaired migration ability found in PDT-derived variants could pass to their progeny with reduced mitochondrial function. Epigenetic mechanisms play crucial roles at the interplay between mitochondrial and nuclear interaction [27]. Therefore, we examined whether DNA methylation and acetylation play an important role in regulating the reduced migration and invasiveness in PDT-derived A375/6A5 variants. To examine the role of DNA methylation in regulating the migration ability, PDT-derived A375/6A5 variants were treated with the methylation inhibitor, 5-azacytidine (5-AZAC, a known DNMT1 inhibitor). As shown in Figure 1A, treatment with 5*-*AZAC did not restore the migration ability of A375/6A5 variants, suggesting DNA methylation might not involve in the reduced migration ability. However, in the presence of HDAC inhibitor trichostatin A (TSA), the migration and invasion abilities of A375/6A5 variants were significantly reverted to that of parental A375 cells (Figure 1B,C). These results indicate that histone hypoacetylation involves in modulating the reduced invasion ability of PDT-derived variants with reduced mitochondrial function. The acetylated modification in the chromatin structure is catalyzed by histone deacetylases (HDACs) and histone acetyltransferases (HATs), which are crucial molecular events in regulating gene expression. Given the above findings, using reverse transcription-PCR (RT-PCR) analysis, we further examined the mRNA expression level of HDAC and HAT in the A375/6A5 variants and parental A375 cells. The results showed that there was no significant difference in the mRNA expression level of the three HATs genes, p300/CBP-associated factor (PCAF), p300, and general control of amino acid synthesis 5 (GCN5) (Figure 1D). However, the expression of HDAC1 and HDAC2 was significantly increased in A375/6A5 variants (Figure 1E). Furthermore, the cellular HDAC enzymatic activity was increased in A375/6A5 variants compared to the parental A375 cells (Figure 1F). The increased HDAC activity in fact correlated with the significant reduction in the acetylated histone H3 in A375/6A5 variant cells (Figure 1G). Together, these findings suggest that histone deacetylation induced by elevated HDAC activity is responsible for the reduced migration and invasiveness in PDT-derived variants with reduced function of mitochondria.

### 2.2. Histone Deacetylation Results in the Down-Regulation of MMP9 and PEG1 in PDT-Derived Variants

**Figure 2:**
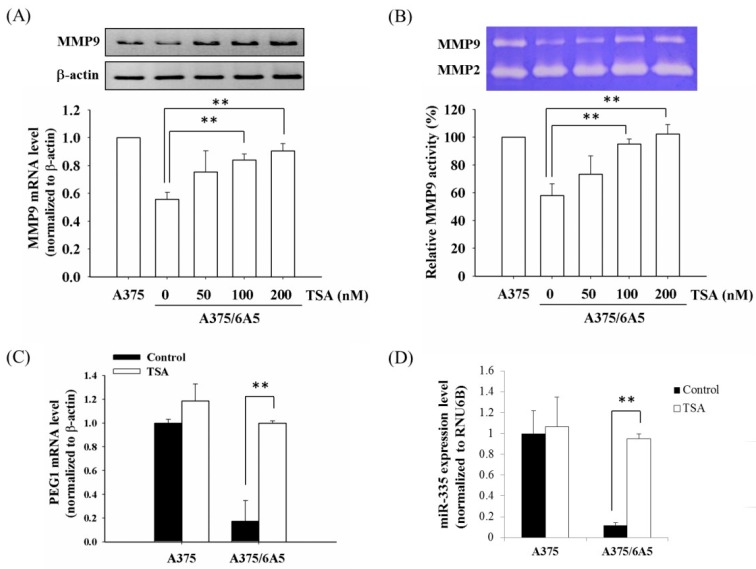
Elevated HDAC activity resulted in the suppressed expression of MMP9 and PEG1 in the PDT-derived variants. (**A**) The mRNA expression of MMP9 was determined in the A375 and A375/6A5 cells 24 h after treated with different concentrations of TSA. **Upper** panel is a represented result of the mRNA amount; **Lower** panel is the average results obtained from three independent experiments (mean ± standard deviation, SD). β-actin served as loading control; (**B**) gelatinase activity of MMP9 was determined in the A375 and A375/6A5 cells 24 h after various TSA treatments. The **upper** panel shows a representative gelatin zymography result; The **lower** panel shows the average of three independent experiments (mean ± SD); (**C**) mRNA expression of PEG1 was analyzed in the A375 and A375/6A5 cells 24 h after treated with 100 nM TSA. β-actin served as loading control; and (**D**) the expression of miR-335 was evaluated in the A375 and A375/6A5 cells 24 h after the treatment with 100 nM TSA. Samples were normalized to the small-nucleolar RNA RNU6B. **, *p* < 0.01.

Using cDNA microarray analysis, two invasion-associated genes, matrix metalloproteinase 9 (MMP9) and paternally expressed gene 1 (PEG1), were down-regulated in the PDT-derived A375 variants. Since histone deacetylase inhibitors (HDACIs) could transcriptionally activate the expression of MMP9 [28], A375/6A5 cells were treated with HDAC inhibitor, TSA, to examine whether the expression of MMP9 and PEG1 was regulated by histone deacetylation. As shown in Figure 2A, the MMP9 expression of A375/6A5 cells was significantly increased in the presence of TSA with a dose-dependent manner. Accordingly, MMP9 gelatin zymography also revealed that the MMP9 gelatinase activity of A375/6A5 cells recovered after TSA treatment (Figure 2B). This result is consistent with a previous study, which reported that the MMP9 expression could be stimulated with TSA in neuroblastoma cells [29].

The PEG1 imprinted gene is expressed only from the paternal allele during development [30]. In cancer cells, the frequent loss of imprinting (LOI) in PEG1 has been reported [31,32]. In addition, the expression of miR-335, a miRNA in the intron 2 region of the PEG1 gene, correlates with the migration or invasion abilities of breast and gastric cancer cells, neuroblastomas, astrocytomas, and bone osteosarcomas [33,34,35,36,37]. It has been proposed that the coordinated expressions of miR-335 and PEG1 might involve in the pathogenesis and progression of numerous cancers [38,39]. However, the regulatory mechanism involved in the expression of PEG1 and miR-335 is still not clear. In this study, PEG-1 was down-regulated in the PDT-derived A375/3A5 variants. Similarly, in the presence of TSA, the expression level of PEG1 and miR-33 cells was also reverted in the A375/6A5 variants, compared to those of A375 parental cells (Figure 2C,D). These results clearly indicate that the expression of PEG1 and miR-335 was regulated by the epigenetic mechanism of histone acetylation in PDT-derived variants with reduced mitochondrial function.

**Figure 3:**
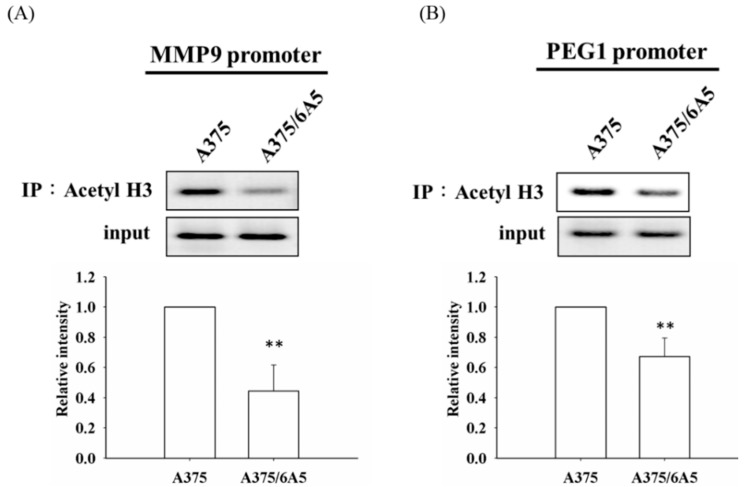
Level of acetylated histone H3 in the promoter of MMP9 gene and PEG1 in PDT-derived variants with mitochondrial dysfunction. Chromatin was harvested from the A375 and PDT-derived A375/6A5 cells, and was precipitated with an anti-acetyl-H3 antibody. The relative amount of bound DNA was evaluated using RT-PCR. PCR amplifications of the (**A**) MMP9 and (**B**) PEG1 promoters from total DNA without prior immunoprecipitation were used as controls for input. A representative experiment from three independent experiments is shown. **, *p* < 0.01.

To address whether the reduction of acetylated H3 (Figure 1G) is responsible for the reduced transcription of MMP9 and PEG1 in the PDT-derived variants, we performed chromatin immunoprecipitation (ChIP) using an antibody specific to acetylated H3 followed by amplifying the promoter region of these two candidate genes. Decreased acetylation of histone H3 was observed in the promoter region of MMP9 and PEG1 in the A375/6A5 variant cells (Figure 3A,B). These results indicate that the reduced expression of MMP9, PEG1, and miR-335 genes relates to the decreased histone acetylation in A375/6A5 variants with reduced mitochondrial function.

Previously, it has been shown that epigenetic modifications by DNA methylation involved in PEG-1 and/or miR-335 expression. The down-regulation of PEG1 by DNA methylation is involved in regulating the development and maternal behavior [30,40,41]. Recently, the coordinated expression of miR-335 and PEG1 was reduced by DNA methylation on their promoter regions in hepatocellular carcinoma [39]. In addition, HDAC3 overexpression was also reported to up-regulate the miR-335 expression in cancer cell lines resistant to microtubule-targeting anti-cancer drugs, which further regulates the invasion, and tumorigenic and angiogenic, responses [42]. In this study, we demonstrated that the increased HDAC1 and HDAC2 expression results in histone deacetylation and further suppress the expression of PEG1 and miR-335 in PDT-derived variants with reduced mitochondrial function.

### 2.3. The Reduced Amount of Acetyl-CoA Also Involved in the Reduced Histone Acetylation and Cellular Migration and Invasiveness in Cells with Mitochondrial Dysfunction

**Figure 4:**
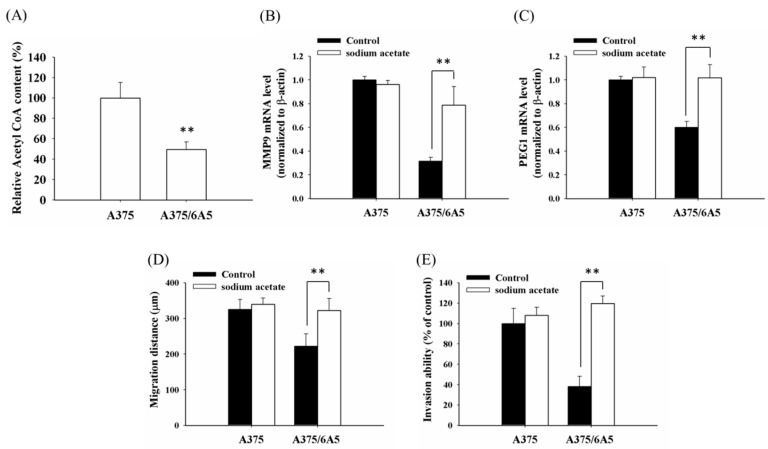
Reduction of acetyl-coenzyme A (acetyl-CoA) content is involved in the mitochondrial dysfunction-mediated histone hypoacetylation in PDT-derived variants. (**A**) Acetyl-CoA content of the A375 and A375/6A5 cells was determined using the PicoProbe™Acetyl-CoA Fluorometric Assay Kit. The mRNA expressions of MMP9 (**B**) and PEG1 (**C**) were determined in A375 and A375/6A5 cells treated with 20 mM sodium acetate. β-actin served as loading control; (**D**) using the scratch wound assay, migration distance (µm) was measured 24 h after treated with 20 mM sodium acetate; and (**E**) after a treatment with 20 mM sodium acetate, the A375 and A375/6A5 cells were allowed to invade through the Matrigel. Invasion of the A375/6A5 cells was normalized to that of parental A375 cells. **, *p* < 0.01.

In addition to the increased HDAC activity, the decrease in histone acetylation might also attribute to the reduced amount of acetyl-CoA pools in mitochondria [43]. As PDT-derived variants have reduced mitochondrial function [7], it is possible that the availability of acetyl-CoA might be limited and subsequently result in the reduction of acetylated histone H3 in the PDT-derived variants. Indeed, the acetyl-CoA content was drastically reduced in the A375/6A5 variants compared to that of A375 parental cells (Figure 4A). To examine whether the reduced amount of acetyl-CoA relates to the repression in invasion and the expression of MMP9 and PEG1 in PDT-derived variants, A375/6A5 cells were treated with sodium acetate to increase the cellular content of acetyl-CoA. As shown in Figure 4B,C, the expression of MMP9 and PEG1 was significantly increased in the A375/6A5 cells with reduced mitochondrial function in the presence of sodium acetate (Figure 4B,C). Meanwhile, the suppressed migration and invasion abilities of PDT-derived variants could be restored (Figure 4D,E). These results suggest that PDT-derived variants with reduced mitochondrial function not only have increased HDAC activity but also have reduced amounts of acetyl-CoA, which further suppressed the expression of MMP9 and PEG1and the following cellular invasiveness.

The role of Ca^2+^ flow changes are often discussed in association with retrograde signaling induced by dysfunctional mitochondria [44,45]. Acetyl-CoA is produced in the mitochondria through the metabolism of fatty acids and the oxidation of pyruvate to acetyl-CoA. As acetyl-CoA is a substrate for histone acetylation, disturbances in acetyl-CoA metabolism might lead to chromatin changes and thus modulate gene expression [46]. Therefore, the decreased acetyl-CoA level might be a retrograde signal that mediates communication between the mitochondria and the nucleus to induce chromatin modifications and control the expression of invasion-related genes in PDT-derived variants with reduced mitochondrial function.

### 2.4. Mitochondrial Dysfunction Induced by 4977 bp Deletion of Mitochondrial DNA and Ethidium Bromide Treatment Suppressed Cellular Migration and Gene Expression of MMP9 and PEG1 via Histone Deacetylation

**Figure 5:**
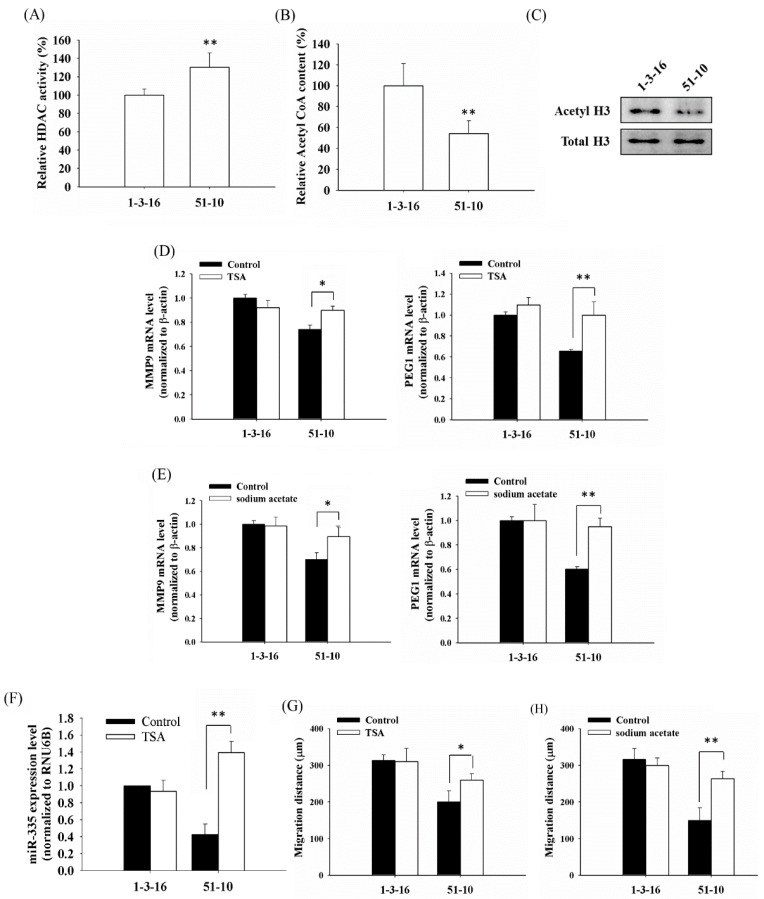
Histone hypoacetylation repressed the expression of MMP9, PEG1 and cellular migration in the cybrids harboring the 4977 bp mtDNA deletion. (**A**) HDAC activities in the nuclear lysates of 1-3-16 and 51-10 cells were determined using a HDAC assay kit; (**B**) Acetyl-CoA content of 1-3-16 and 51-10 cells were determined using the PicoProbe™Acetyl-CoA Fluorometric Assay Kit; (**C**) Western blot analysis of acetylated histone H3 in the 1-3-16 and 51-10 cells. mRNA expression of MMP9 and PEG1 was determined in the 1-3-16 and 51-10 cells 24 h after the treatment with 100 nM TSA (**D**) or 20 mM sodium acetate (**E**). β-actin served as loading control; (**F**) the expression of miR-335 was evaluated in the 1-3-16 and 51-10 cells 24 h after the treatment with 100 nM TSA. Samples were normalized to the small-nucleolar RNA RNU6B. Using the scratch wound assay, the migration distance (µm) was measured 24 h after the treatment with 100 nM TSA (**G**) or 20 mM sodium acetate (**H**). *, *p* < 0.05; **, *p* < 0.01.

To verify the repressed invasion and expression of MMP9 and PEG1 by histone deacetylation is not PDT-derived variants specific, we analyzed other cellular types with dysfunctional mitochondria induced by different modes of treatment. To this end, we used another cell lines, 51-10 cybrid cells with approximately 85% of the 4977 bp mtDNA deletion, which were generated by fusing the mtDNA-less 143B osteosarcoma (ρ^0^) cells with enucleated skin fibroblasts from a patient with clinically proven chronic progressive external ophthalmoplegia (CPEO) [47]. As shown in Figure 5A, the HDAC activity in the 51-10 cybrid cells was significantly higher, compared to the wild-type 1-3-16 cells with intact mtDNA (Figure 5A). In addition, the acetyl-CoA content of the 51-10 cybrid cells was significantly lower than that of the 1-3-16 cells (no deletion in mtDNA), and accordingly, acetylated H3 level was decreased in 51-10 cybrid cells with the 4977 bp mtDNA deletion (Figure 5B,C). Furthermore, the expression of MMP9 and PEG1 were decreased in the Δ4977 cybrid 51-10 cells compared to that of 1-3-16 wild-type cells. In the presence of TSA or sodium acetate, the expression level of MMP9 and PEG1 was significantly elevated in the mtDNA-deletion 51-10 cells (Figure 5D,E). Similar results were also found in the expression of miR-335 in 51-10 cells (Figure 5F). Furthermore, the migration ability was restored in the Δ4977 cybrid following TSA or sodium acetate treatment (Figure 5G,H).

**Figure 6:**
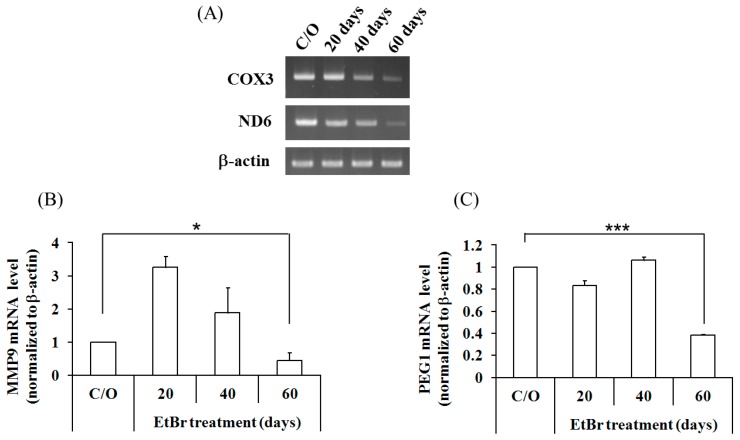
MMP9 and PEG1 were down-regulated in the A375 ρ^0^ cells. (**A**) A375 cells were cultured in a medium containing ethidium bromide (EtBr) for 20, 40, or 60 days, and transcription levels of mitochondrial genes were measured with RT-PCR using primers that specifically target cytochrome oxidase c III (COX3) and NADH dehydrogenase 6 (ND6). Similarly, expression of MMP9 (**B**) and PEG1 (**C**) was determined. β-actin served as loading control. *, *p* < 0.05; ***, *p* < 0.001.

A375/6A5 variants were established as stable clones from human melanoma A375 cells after consecutive ALA-mediated photodynamic treatment. In contrast, 51-10 cells were generated by fusing the mtDNA-less 143B osteosarcoma (ρ^0^) cells with enucleated skin fibroblasts from a CPEO patient [47]. In A375/6A5 variant cells, the reduced mitochondrial function relates to the accumulated damage by PDT-mediated oxidative stress. However, the mitochondrial dysfunction in 51-10 cells is attributed to the 4977 bp mtDNA deletion (approximately 85%). In these two cell lines, mitochondrial dysfunction was induced by a different mode of treatment; however, they all have reduced histone acetylation and further result in the suppressed cellular migration/invasion and expression of MMP9, PEG1, and miR-335. In this regard, we argue that mitochondria might play an important role in modulating the expression of invasion-related genes through histone acetylation. To further verify this, a mitochondria-depleted cell line, A375 ρ^0^, was generated following long-term exposure to ethidium bromide (EtBr). In the established A375 ρ^0^ cells, we found a substantial decrease in the transcription level of mitochondria-encoded cytochrome c oxidase III (COX3) and mitochondria-encoded NADH dehydrogenase 6 (ND6) without changes in the transcription of nuclear housekeeping genes (Figure 6A). As shown in Figure 6B,C, 60 days of EtBr treatment (long-term culture with >40 passages) resulted in the decreased expression of MMP9 and PEG1, suggesting the decreased expression of MMP9 and PEG1 might be a general phenomenon in cells with dysfunctional mitochondria.

## 3. Experimental Section

### 3.1. Cell Culture

The PDT-derived variant of A375 (A375/6A5) was established by treating the parental A375 cell lines with ALA-PDT as described previously [7]. The A375 and A375/6A5 cells were cultured in DMEM medium supplemented with 10% fetal bovine serum (FBS) at 37 °C in a humidified atmosphere of 5% CO_2_ and 95% air.

The wild-type 1-3-16 cells (no deletion in mtDNA) and the mutant 51-10 cells (the 4977 bp mtDNA deletion) were kindly provided by Yau-Huei Wei [47]. The 51-10 and 1-3-16 cells were grown in DMEM supplemented with 5% FBS, 100 µg/mL pyruvate, 50 µg/mL uridine, and antibiotics composed of 100 U/mL penicillin G and 100 µg/mL streptomycin sulfate (Gibco^®^, Life Technologies Corporation, Carlsbad, CA, USA) at 37 °C in a humidified atmosphere of 5% CO_2_/95% air.

To deplete mtDNAs in the A375 cells, long-term culture (>40 passages) was conducted using a medium containing 50 ng/mL ethidium bromide (Sigma, St. Louis, MO, USA). The mtDNA-depleted sub-line was conventionally designated as A375 ρ^0^ cells.

### 3.2. RNA Extraction, mRNA and miRNA Detection

After various treatments, total RNA was extracted from the cells with the TRIzol^®^ Reagent (Invitrogen™, Life Technologies Corporation, Carlsbad, CA, USA). For each sample, the amount of total RNA was measured, and 1 µg RNA was reverse transcribed into cDNA using random hexamers. The resulting cDNA was amplified with PCR (reaction mixture: 2.5 U Taq DNA polymerase, 3 mM MgCl_2_, 1× PCR buffer, 0.4 mM dNTP, and the respective specific primers listed in Table 1). β-actin was used as internal control. PCR products were loaded on a 1.5% agarose gel in Tris-acetate-EDTA (TAE) buffer, and DNA was visualized with EtBr. To analyze the expression of miR-335, total RNA was isolated from the cultured cells using the mirVana miRNA Isolation Kit (Ambion^®^, Life Technologies Corporation, Carlsbad, CA, USA). Mature form of the miRNA was detected using the mirVana qRT-PCR miRNA Detection Kit and the qRT-PCR Primer Sets specific for miR-335, according to the manufacturer’s instructions (Ambion^®^, Life Technologies Corporation). U6 small nuclear RNA was used as internal control.

### 3.3. Western Blot

Protein samples were prepared from cells under different treatments. Proteins were separated using 12% SDS-PAGE, which were further blotted onto nitrocellulose membranes. Membranes were incubated with antibodies against acetyl-histone H3 (Millipore, Temecula, CA, USA) and histone H3 (GeneTex, Irvine, CA, USA). Then, the secondary antibody conjugated with horseradish peroxidase was added, and immunoreactivity was detected using chemiluminescence.

### 3.4. Migration and Invasion Assays

For transwell migration assays, 2 × 10^4^ cells were plated in the top chamber with a non-coated membrane (24-well insert; pore size: 8 µm; BD Biosciences, San Jose, CA, USA). For invasion assays, 2 × 10^4^ cells in serum free medium were seeded onto the top chamber with a Matrigel-coated membrane (24-well insert; pore size: 8 µm; BD Biosciences). In the lower chamber, the medium contains 10% FBS was used as a chemo-attractant. After 24 h incubation, a cotton swab was used to remove the cells that did not invade through the membrane pores. Cells on the lower surface of the membrane were counted after staining with 0.1% crystal violet (Sigma).

**Table 1:** List of primers used for amplification using RT-PCR.

Gene Name	Primer
*PCAF* (forward)	5′-TCCTGTCGGAGTTGTAGCCA-3′
*PCAF* (reverse)	5′-GTTCTGGAAGAGGCTGAGAG-3′
*p300* (forward)	5′-AGCCCTGGCAGTATGTCGAT-3′
*p300* (reverse)	5′-GAATCCAGCAGGCCAGATGA-3′
*GCN5* (forward)	5′-GTCAGGCTTCACCATGCCAC-3′
*GCN5* (reverse)	5′-TGCCGATGACATGGAACTCG-3′
*HDAC1* (forward)	5′-AACCTGCCTATGCTGATGCT-3′
*HDAC1* (reverse)	5′-CAGGCAATTCGTTTGTCAGA-3′
*HDAC2* (forward)	5′-GGGAATACTTTCCTGGCACA-3′
*HDAC2* (reverse)	5′-ACGGATTGTGTAGCCACCTC-3′
*HDAC3* (forward)	5′-TGGCTTCTGCTATGTCAACG-3′
*HDAC3* (reverse)	5′-GCACGTGGGTTGGTAGAAGT-3′
*MMP9* (forward)	5′-CGGAGCACGGAGACGGGTAT-3′
*MMP9* (reverse)	5′-TGAAGGGGAAGACGCACAGC-3′
*PEG1* (forward)	5′-CAAAGATGGAGGTGTGC-3′
*PEG1* (reverse)	5′-TTCCCGTCATTGTTGCG-3′
*β-actin* (forward)	5′-TGGACTTCGAGCAAGAGATGG-3′
*β-actin* (reverse)	5′-ATCTCCTTCTGCATCCTGTCG-3′
*ND6* (forward)	5′-CCCGAGCAATCTCAATTACA-3′
*ND6* (reverse)	5′-CCGTGCGAGAATAATGATGT-3′
*COX3* (forward)	5′-AGCCATGTGATTTCACTTCC-3′
*COX3* (reverse)	5′-GTTGAGCCAATAATGACGTG-3′

### 3.5. Chromatin Immunoprecipitation (ChIP) Assay

Confluent cells on 10 cm plates were treated with 1% formaldehyde in phosphate buffered saline for 10 min at 37 °C. Chromatin was immunoprecipitated using a polyclonal antibody against acetyl-histone H3 (Millipore, Temecula, CA, USA). Briefly, sonication of the crosslinked nuclei yielded DNA fragments in the range of 200–1000 bps. Anti-histone H3 antibodies were incubated overnight with the precleared nuclear lysates. Immune complexes were then collected with protein A magnetic beads (Millipore). The recovered DNA was amplified with PCR. PCR products were visualized by gel electrophoresis to check the amount and correct size of the DNA products (see Table 2 for the list of primers used for the ChIP assay). Each ChIP experiment was repeated at least three times.

**Table 2:** List of primers used for chromatin immunoprecipitation (ChIP).

Gene Name	Primer
*MMP9* promoter (forward)	5′-CTGAGTCAAAGAAGGCTGT-3′
*MMP9* promoter (reverse)	5′-GTGATGGAAGACTCCCTGAGA-3′
*PEG1* promoter (forward)	5′-TACAGACTAGAGAAGGAAGCG-3′
*PEG1* promoter (reverse)	5′-CGTGCTCTCGATCCTTATATT-3′
*EGFR* promoter (forward)	5′-ACCCTGGCACAGATTTGG-3′
*EGFR* promoter (reverse)	5′-TGAGGAGTTAATTTCCGAGAGG-3′

### 3.6. MMP9 Activity

MMP9 gelatinase activity was assessed by gelatin zymography. Cells treated with TSA or sodium acetate were incubated in DMEM medium without serum for 24 h. The conditioned medium was collected from each treatment group and was then centrifuged (1000 rpm, 5 min) to remove cells and cell debris. The clear supernatants were subjected to SDS-PAGE without boiling or reduction. The gels contained 10% polyacrylamide copolymerized with 0.1% gelatin. After electrophoresis, gels were washed twice for 30 min in 2.5% Triton X-100, and then were incubated at 37 °C for 18 h in the incubation buffer (50 mM Tris, 10 mM CaCl_2_, 0.2 M MgCl, and 0.02% Brij-35; pH 8). After incubation, gels were stained in 0.5% Coomassie Brilliant Blue R-250 (Sigma) for 60 min, and then washed in the destaining solution (25% ethanol and 8% acetic acid). Gelatinolytic activity was represented by a clear band against the blue background of the gel. Digestive activity of MMP9 was confirmed by the presence of a 94-kDa band on the zymogram using a prestained protein marker as reference.

### 3.7. HDAC Activity

HDAC activity was analyzed with an HDAC activity colorimetric assay kit (BioVision, Mountain View, CA, USA) according to the manufacturer’s instructions. Briefly, each nuclear extract was mixed with the HDAC colorimetric substrate in the HDAC assay buffer for 2 h at 37 °C. The reaction was terminated by adding the Lysine Developer and incubating the plate at 37 °C for 30 min. Absorbance was measured at 405 nm using an ELISA plate reader.

### 3.8. Acetyl-CoA Content

Acetyl-CoA content was analyzed with the PicoProbe™Acetyl-CoA Fluorometric Assay Kit (BioVision, Mountain View, CA, USA) according to the manufacturer’s instructions. Before performing the assay, samples were deproteinized using the Deproteinizing Sample Preparation Kit (BioVision) to avoid enzymatic interference. Then, samples were mixed with 50 µL reaction mixture (Acetyl CoA Assay Buffer, Acetyl CoA Substrate Mix, Conversion Enzyme, Acetyl CoA Enzyme Mix, and PicoProbe), and the plate was incubated at 37 °C for 10 min. Fluorescence was measured using a fluorescence spectrometer (excitation/emission: 535/589 nm).

### 3.9. Statistical Analysis

Statistical analysis was conducted using one-way analysis of variance followed by Dunnett’s *post hoc* test using the GraphPad InStat software, version 3.00 for Windows (GraphPad Software, San Diego, CA, USA).

## 4. Conclusions

Compared to parental cells, the elevated HDAC activity and reduced amount of acetyl-CoA result in the suppressed invasiveness, which correlate with the down-regulation of MMP9, PEG1, and miR-335 in A375/6A5 variants with reduced function of mitochondria. The effect of mitochondrial dysfunction on invasiveness and gene expression are further confirmed in cells with the 4977 bp deletion of mitochondrial DNA or mitochondria depletion, indicating histone acetylation might play an important role in regulating migration and invasiveness in cells containing dysfunctional mitochondria.
